# Intracoronary Sarcoplasmic Reticulum Calcium-ATPase Gene Therapy in Advanced Heart Failure Patients with reduced Ejection Fraction: A Prospective Cohort Study

**DOI:** 10.6061/clinics/2020/e1530

**Published:** 2020-03-09

**Authors:** Jianfeng Zhang, Guojin Hu, Shengyong Yang

**Affiliations:** Cardio-Pulmonary Rehabilitation Inpatient Area, The Second Rehabilitation Hospital of Shanghai, Shanghai 200431, China

**Keywords:** Cardiac Events, Ejection Fraction, Heart Failure, Intracoronary Delivery, Sarcoplasmic Reticulum calcium-ATPase Gene Therapy

## Abstract

**OBJECTIVE:**

Heart failure is a progressive and debilitating disease. Intracoronary sarcoplasmic reticulum calcium-ATPase gene therapy may improve the function of cardiac muscle cells. This study aimed to test the hypothesis that intracoronary sarcoplasmic reticulum calcium-ATPase gene therapy can improve outcomes and reduce the number of recurrent and terminal events in advanced heart failure patients with reduced ejection fraction.

**METHODS:**

A total of 768 heart failure patients with reduced ejection fraction and New York Heart Association classification II to IV were included in this prospective cohort study. Patients either underwent intracoronary sarcoplasmic reticulum calcium-ATPase gene therapy (CA group, n=384) or received oral placebo (PA group; n=384). Data regarding recurrent and terminal event(s), treatment-emergent adverse effects, and outcome measures were collected and analyzed.

**RESULTS:**

After a follow-up period of 18 months, intracoronary sarcoplasmic reticulum calcium-ATPase gene therapy reduced the number of hospital admissions (*p*=0.001), ambulatory treatments (*p*=0.0004), and deaths (*p*=0.024). Additionally, intracoronary sarcoplasmic reticulum calcium-ATPase gene therapy improved the left ventricular ejection fraction (*p*<0.0001) and Kansas City Cardiomyopathy Questionnaire score (*p*<0.0001). The number of recurrent and terminal events/patients were higher in the PA group than in the CA group after the follow-up period of 18 months (*p*=0.015). The effect of the intracoronary sarcoplasmic reticulum calcium-ATPase gene therapy was independent of the confounding variables. No new arrhythmias were reported in the CA group.

**CONCLUSIONS:**

Intracoronary sarcoplasmic reticulum calcium-ATPase gene therapy reduces the number of recurrent and terminal events and improves the clinical course of advanced heart failure patients with reduced ejection fraction.

## INTRODUCTION

Heart failure is a progressive and debilitating disease (1). It is associated with inadequate contractility of the heart (2) due to abnormal calcium cycling (3). Morbidity and mortality in heart failure patients are high (4,5). Drugs for heart failure only slow down the progression of the disease but do not cure the disease (6). Calcium-ATPase deficiency is generally associated with the progression of heart failure (7,8). During diastole, the sarcoplasmic reticulum calcium-ATPase regulates contraction and relaxation of cardiac muscle cells by transporting calcium from the cytosol into the sarcoplasmic reticulum (9). Gene therapy restores the function of the heart as a pump (2). If calcium-ATPase deficiency is corrected, the function of the cardiac muscle cells may be improved in heart failure patients (7).

An experimental model (7,8), a phase 2 trial involving a high-dose of sarcoplasmic reticulum calcium-ATPase gene therapy in advanced heart failure patients (10), and a randomized trial (3) involving the use of gene therapy for the treatment of cardiac disease have confirmed the hypothesis that sarcoplasmic reticulum calcium-ATPase gene transfer improves survival and the performance of cardiac muscle cells in heart failure conditions. However, a phase 2b trial involving high-risk ambulatory patients with heart failure (9) revealed that high-dose intracoronary sarcoplasmic reticulum calcium-ATPase gene therapy does not improve cardiac muscle cell performance in heart failure patients. Thus, the favorable effect of targeted intracoronary sarcoplasmic reticulum calcium-ATPase gene therapy in heart failure patients has not been thoroughly investigated.

The aim of this study is to test the hypothesis that intracoronary sarcoplasmic reticulum calcium-ATPase gene therapy can improve outcomes and reduce the number of recurrent and terminal events in advanced heart failure patients with reduced ejection fraction.

## MATERIALS AND METHODS

### Ethics consideration and consent to participate

The designed protocol (SRHS/CL/12/15 dated December 5^th^ 2015) of the established study was approved by the review board of Second Rehabilitation Hospital of Shanghai. The study was reported according to the laws of China, strengthening the reporting of observational studies in epidemiology (STROBE) statement, and the 2008 Helsinki Declaration. An informed consent form was signed by all enrolled patients regarding the publication of data on pathology and interventions, including personal data and images (if any), in all formats (hard and/or electronic).

### Inclusion criteria

Patients aged 18 to 80 years, who had experienced chronic heart failure as per the 2016 European Society of Cardiology (ESC) Guidelines (11) (confirmed by left ventricular angiography for ejection fraction), and had been undergoing medical treatment for at least one month in the Second Rehabilitation Hospital of Shanghai, Shanghai, China, were considered for the study. Among these patients, only those with the New York Heart Association (NYHA) heart functional classification II to IV (for ischemic or non-ischemic etiology) and a left ventricular ejection fraction 35% or less were included in the analysis. Heart failure patients with undetectable neutralizing antibodies who could not block vector entry of adeno-associated viruses 1 into the target cells were only included in the study.

### Exclusion criteria

Patients aged above 80 years, pregnant females, those who had undergone cardiac surgery, and those who had undergone either percutaneous coronary intervention, acute heart failure treatment (positive inotropes, intravenous vasodilators, or diuretics), or valvuloplasty were excluded from the study. Restrictive cardiomyopathic patients, obstructive cardiomyopathic patients, and patients who had amyloidosis (confirmed by blood and urine tests, biopsies of belly fat, and imaging methods), acute myocarditis, infiltrative cardiomyopathy, pericardial disease, thyroid disease, abnormal liver function, anemia, thrombocytopenia, carcinoma, sarcoma, and/or cancer were also excluded from the study.

### Sample size calculation

The sample size was calculated using OpenEpi (Epidemiologic Statistics for Public Health, USA) at a power of 80%. The sample size for both cohorts was calculated as 384.

### Cohorts

Patients who received intracoronary 1×10^13^ DNase-resistant particles of adeno-associated virus 1 sarcoplasmic reticulum calcium-ATPase (CA) (Luxturna; Sparks Therapeutics, Philadelphia, PA, USA) were assigned to the CA group (n=384) and patients who received oral placebo (Cebocap, Forte Pharmaceutical, Hyderabad, India) were assigned to the PA (placebo group; n=384) (9). Data on the function of the liver, kidney, and other vital organs were reviewed before the administration of calcium-ATPase.

### Recurrent and terminal events during a follow-up period of 18 months

The number of hospital admissions due to heart failure and the number of ambulatory treatments for worsening conditions were acquired. Information regarding all causes of death, heart transplantations performed, and the use of a mechanical ventilation system were also collected (6). Additionally, data on the incidences and severity of interventions-emergent adverse events and the number of deaths (cardiovascular-related) were collected (9).

### Outcome measures

We collected data on blood pressure, changes in the NYHA functional class of the heart, Kansas City Cardiomyopathy Questionnaire (KCCQ; 23-item questionnaire; the score ranged from 0 to 100) score (12), exercise ability (6-min walk test; comparison was made with patients who had no neuromuscular, orthopedic, or rheumatologic abnormality, and were able to walk 300–400 m in 6 min (6)), creatinine level (10), and N-terminal pro-B-type natriuretic peptide (NT-proBNP) at baseline and at 1, 3, 6, 10, and 18 months after interventions. Endomyocardial biopsy was performed after 18 months of intervention to evaluate the development of new heart failure and/or arrhythmias (13).

Outcome measures were collected by physicians and the nursing staff of the institute(s) (all personnel had a minimum of three years of experience). For patients who died during the follow-up period, the last reported data were used for analysis.

### Safety

All new arrhythmias found during the 18-month follow-up period were recorded.

### Statistical analysis

For statistical analysis, InStat, 3.1 Window, GraphPad, San Diego, CA, USA was used. For ordinal data, the Chi-square Independence test (4) was used for statistical analysis while the one-way analysis of variance (ANOVA) (14) was used to analyze continuous variables. Logistic regression analysis was performed to evaluate the risk factors for recurrent and terminal events during the 18-month follow-up period. All results were considered significant at a 95% confidence level.

## RESULTS

### Clinical characteristics

Among the enrolled patients, 80% were males and 99% were Han Chinese. Additionally, most of the patients had NYHA heart functional class III. The other clinical characteristics of the patients are presented in Table 1. At baseline, both groups had the same demographical and clinical characteristics (*p*>0.05 for all).

### Recurrent and terminal events

**Figure 1:**
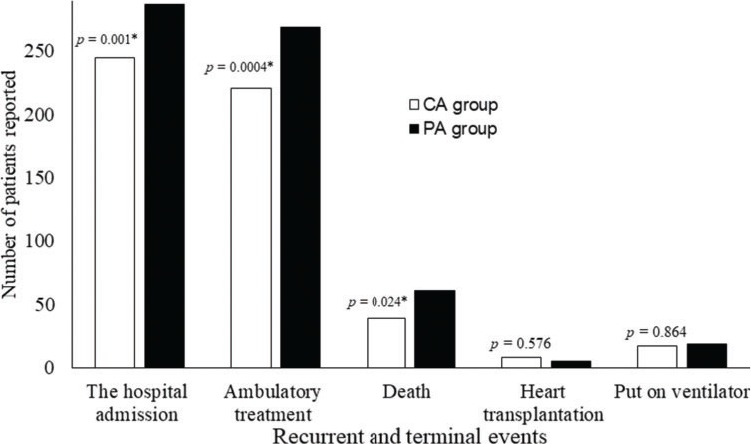
Recurrent and terminal events after a follow-up period of 18 months. The chi-square test of independence was performed between the two groups. A *p*-value <0.05 was considered significant. *A significant fewer values reported than the placebo group.

After a follow-up period of 18 months, intracoronary sarcoplasmic reticulum calcium-ATPase gene therapy reduced the number of hospital admissions (*p*=0.001), ambulatory treatments (*p*=0.0004), and deaths (*p*=0.024) but was not successful in reducing the number of heart transplantations (*p*=0.576) and mechanical ventilation incidences (*p*=0.864, Figure 1).

### Outcome measures

After a follow-up period of 18 months, intracoronary sarcoplasmic reticulum calcium-ATPase gene therapy improved the heart function (*p*=0.009), left ventricular ejection fraction (*p*<0.0001), KCCQ score (*p*<0.0001), systolic blood pressure (*p*<0.0001), and performance in the 6-min walk test (*p*=0.047). However, intracoronary sarcoplasmic reticulum calcium-ATPase gene therapy failed to reduce the NT-proBNP (*p*=0.482) and serum creatinine (*p*=0.822, Table 2) levels.

**Figure 2:**
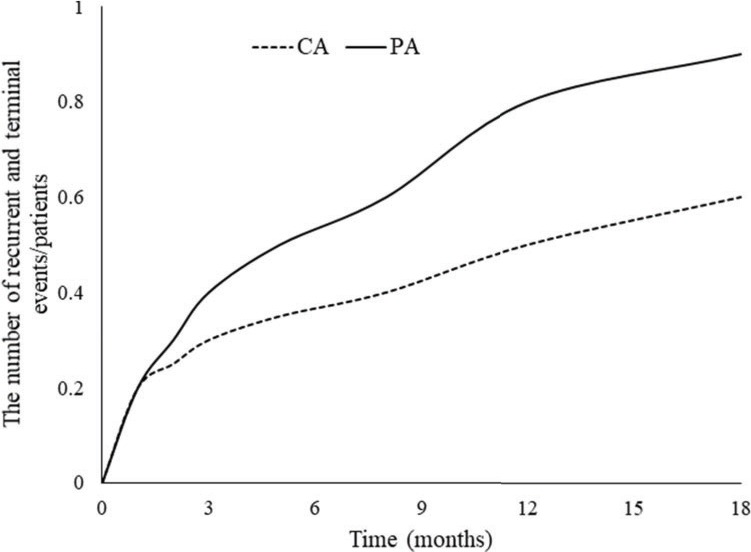
Recurrent and terminal events after a follow-up period of 18 months.

Age (*p*=0.048), NT-proBNP (*p*=0.046), and placebo treatment (*p*=0.021) were associated with recurrent and terminal events during the 18-month follow-up period and the effect of the intracoronary sarcoplasmic reticulum calcium-ATPase gene therapy was independent of the confounding variables (Table 3). Moreover, cumulative recurrent and terminal events/patients were higher in the PA group than in the CA group during the 18-month follow-up period (*p*=0.015, Figure 2).

### Safety

In the CA group, endomyocardial biopsies performed after the 18-month follow-up period, revealed no new arrhythmias or treatment-emergent adverse effects.

## DISCUSSION

In the present study, intracoronary sarcoplasmic reticulum calcium-ATPase gene therapy reduced the number of recurrent and terminal events. In heart failure patients, a decrease in sarcoplasmic reticulum calcium-ATPase activity results in reduced calcium uptake during relaxation (6), which is associated with depressed calcium homeostasis and reduced cardiomyocyte function (9) due to the reduction in the contractile function of the heart (5). Recurrent and terminal events are frequent during the clinical course of patients with cardiac disease (15) due to disturbance of the immune pathway and the short duration of transgene expression (16), which leads to increases in the financial burden of patients (17). The correction of these abnormalities using intracoronary DNase-resistant particles of adeno-associated virus 1 can improve cardiac function and the survival rate (8,18) by improving vascular reactivity and coronary flow (19). Additionally, intracoronary calcium-ATPase transfer decreases the number of ventricular arrhythmias and improves the arrhythmogenic substrate and the factors that trigger it by entering the cardiac cells, where high transduction efficiency is necessary. Moreover, entering of the adeno-associated viruses 1 in cardiac cells tropism provides homogeneous cardiac myocyte transduction (3). The results of the analysis were consistent with the results of previous experimental studies (16,18), pilot studies (3,9), and a phase 2 trial (10), but were not consistent with the results of the phase 2b trial (9). The results of the current study showed that the intracoronary DNase-resistant particles of adeno-associated virus 1 sarcoplasmic reticulum calcium-ATPase may improve the clinical course of heart failure patients with reduced ejection fraction.

Intracoronary sarcoplasmic reticulum calcium-ATPase gene therapy improved the NYHA class, performance during the 6-min walk test, left ventricular ejection fraction, KCCQ score, and systolic blood pressure. The improvement in the KCCQ score and 6-min walk test would result in an increase in the ability of patients to perform physical activities, which would result in the improvement of the NYHA class (20). Intracoronary sarcoplasmic reticulum calcium-ATPase gene therapy significantly inhibits left ventricular dilation, which restores systolic functions of the heart (21). The results of the analysis were consistent with the results of the phase 2 trial (10) and experimental studies (16,22). The positive results regarding outcome measures showed that intracoronary sarcoplasmic reticulum calcium-ATPase gene therapy may be beneficial to heart failure patients with reduced ejection fraction.

Intracoronary sarcoplasmic reticulum calcium-ATPase gene therapy failed to reduce the NT-proBNP (*p*=0.482) and creatinine (*p*=0.822) levels. An NT-proBNP level higher than 1600 pg/mL is responsible for recurrent events (6). The results of the analysis were consistent with the results of another study (9). NT-proBNP released as a result of targets not achieved by intracoronary sarcoplasmic reticulum calcium-ATPase gene therapy (23). In addition, it is well known that the hydrosaline retention state present during heart failure is related not only to the hemodynamic phenomenon due to the heart pumping failure but also as a result of various neurohumoral mechanisms that maintain a reduced glomerular filtration rate, which would remain constant regardless of the improvement in pump function after intracoronary sarcoplasmic reticulum calcium-ATPase gene therapy (24). A negative outcome may slow down, but not deter, further research. These negative results need to be addressed in future randomized trials.

Intracoronary sarcoplasmic reticulum calcium-ATPase gene therapy improved the clinical course of the enrolled patients without any treatment-emergent adverse effects. Our results are consistent with those of published studies (3,9,10). Our positive results show that intracoronary sarcoplasmic reticulum calcium-ATPase gene therapy is safe and promising.

In this study, gene therapy was performed via intracoronary delivery. The percutaneous, cardiac-perfusion circuit enhances the uptake of the gene by cardiomyocytes (16). However, intracoronary delivery is simple, practical, and also enhances uptake of the gene (5). We recommend intracoronary delivery for gene therapy for optimal effect.

In this study, we reported recurrent and terminal events simultaneously. Each recurrent event in patients increases the risk of additional recurrent and terminal events (25). For example, the mortality rate is higher after the fourth hospitalization compared to that after the first hospitalization (6). Thus, to avoid bias, we evaluated recurrent and terminal events simultaneously.

One limitation of the study is its prospective nature and the lack of a randomized trial. If a patient died during the follow-up period, the last reported data were used for analysis. This increases the possibility of the occurrence of bias. We did not evaluate the bias between coronary intervention and oral administration.

## CONCLUSION

Intracoronary sarcoplasmic reticulum calcium-ATPase gene therapy reduces the number of recurrent and terminal events and improves the clinical course of advanced heart failure patients with reduced ejection fraction.

## AUTHOR CONTRIBUTIONS

All authors read and approved the manuscript for publication. Zhang J contributed to formal analyses, resources, and literature review, and was responsible for the manuscript drafting, review and edition for intellectual content. Hu G was the project administrator and contributed to software development, formal analyses, validation, and literature review. Yang S contributed to resources, data curation, investigation, software development, and literature review. The authors agree to be accountable for all aspects of the work.

## Figures and Tables

**Table 1:** Demographic and clinical characteristics of the enrolled patients.

Characteristics		Groups		Comparison between groups
		CA	PA	
Heart failure patients enrolled in the cohort		384	384	
Treatment		Intracoronary sarcoplasmic reticulum Calcium-ATPase gene therapy	Oral placebo	*p*-value
Age (years)	Minimum	18	18	0.272
	Maximum	80	80	
	Mean ± SD	59.12±11.45	60.11±13.45	
Gender	Male	308 (80)	306 (80)	0.928
	Female	76 (20)	78 (20)	
Ethnicity	Han Chinese	380 (99)	379 (98.7)	0.930
	Tibetan	1 (0.3)	1 (0.3)	
	Mongolian	3 (0.7)	4 (1)	
6-min walk test (m)		315.15±49.47	321.52±61.52	0.114
Left ventricular ejection fraction (%)		24.12±4.15	24.92±7.15	0.058
NYHA heart functional classification	II	75 (20)	74 (19)	0.996
	III	301 (78)	302 (79)	
	IV	8 (2)	8 (2)	
KCCQ score		60.12±3.15	59.68±4.01	0.09
NT-proBNP (pg/mL)		1511±102	1499±99	0.099
Cause of heart failure	Idiopathic	160 (42)	162 (42)	0.970
	Ischemic	194 (50)	191 (50)	
	Hereditary	4 (1)	5 (1)	
	Hypertension	18 (5)	20 (5)	
	Peripartum	8 (2)	6 (2)	
Abnormal renal function		8 (2)	9 (2)	0.806
Chronic obstructive pulmonary disease		15 (4)	11 (3)	0.550
Creatinine (mg/dL)		1.81±0.61	1.79±0.59	0.644
Systolic blood pressure (mmHg)		132±6	133±8	0.051
Medical treatment	Special beta-blockers	155 (41)	165 (43)	0.801
	Angiotensin-converting enzyme inhibitors	101 (26)	99 (26)	
	Angiotensin II receptor antagonist	85 (22)	75 (19)	
	Aldosterone receptor antagonists	43 (11)	45 (12)	

NYHA: New York Heart Association.

KCCQ: Kansas City Cardiomyopathy Questionnaire (range: 0 to 100).

NT-proBNP: N-terminal pro-B-type natriuretic peptide (NT-proBNP level <1600 pg/mL was considered as normal).

Ordinal data are shown as frequency (percentage) and continuous variable are shown as mean ± SD.

The Chi-square Independence test for ordinal data and one-way ANOVA for continuous variables were used for statistical analyses.

A *p*-value <0.05 was considered significant.

**Table 2:** Outcome measures for cardiac function reported after a follow-up period of 18 months.

Characteristics		Groups						Comparison between groups at EL
		CA			PA			
Treatment		Intracoronary sarcoplasmic reticulum Calcium-ATPase gene therapy			Oral placebo			
Level		BL	EL	SA	BL	EL	SA	
Patients enrolled in the cohort		384	384	*p*-value	384	384	*p*-value	*p*-value
NYHA heart functional classification	II	75 (20)	105 (27)	0.023	74 (19)	73 (19)	0.891	0.009
	III	301 (78)	275 (72)		302 (79)	301 (78)		
	IV	8 (2)	4 (1)		8 (2)	10 (3)		
6-min walk test (m)		315.15±49.47	325.27±51.52	0.006	321.52±61.52	323.47±65.47*	0.671	0.047
Left ventricular ejection fraction (%)		24.12±4.15	29.35±7.45	<0.0001	24.92±7.15	25.12±7.65*	0.708	<0.0001
KCCQ score		60.12±3.15	65.45±5.46	<0.0001	59.68±4.01	60.01±4.45*	0.281	<0.0001
§NT-proBNP (pg/mL)		1511±102	1501±99*	0.168	1499±99	1496±98*	0.673	0.482
Creatinine (mg/dL)		1.81±0.61	1.79±0.62*	0.652	1.79±0.59	1.78±0.6*	0.817	0.822
Systolic blood pressure (mmHg)		132±6	128±5	<0.0001	133±8	132±6*	0.051	<0.0001

BL: At the time of enrollment.

EL: After a follow-up period of 18 months.

SA: Statistical analysis between BL and EL.

Ordinal data are shown as frequency (percentage) and continuous variable are shown as mean ± SD.

The Chi-square Independence test for ordinal data and one-way ANOVA for continuous variables were used for statistical analyses.

A *p*-value <0.05 was considered significant.

If the patient died during the follow-up period, the last reported data were used for analysis.

* Insignificant difference with respect to BL.

§ <1600 pg/mL was considered normal.

**Table 3:** The influence of risk factors on recurrent and terminal events after a follow-up period of 18 months.

Heart failure patients included in the analysis		768		
Characteristics		Risk ratio	95% CI	*p*-value
*Age (years)		4.05	0.75-4.61	0.048
Gender		0.56	0.42-1.12	0.82
Ethnicity		0.54	0.53-1.15	0.63
6-min walk test (m)		0.68	0.54-1.17	0.65
Left ventricular ejection fraction (%)		0.42	0.67-1.19	0.68
NYHA heart functional classification		0.46	0.56-1.09	0.67
KCCQ score		0.52	0.52-1.08	0.56
*NT-proBNP (pg/mL)		3.87	0.81-4.22	0.046
Cause of heart failure		0.54	0.82-1.12	0.56
Abnormal renal function		0.55	0.63-1.15	0.63
Chronic obstructive pulmonary disease		0.63	0.68-1.27	0.59
Creatinine (mg/dL)		0.71	0.56-1.41	0.63
Treatment	Calcium-ATPase	0.82	0.51-0.98	0.82
	*Placebo	6.52	1.12-6.12	0.021

NYHA: New York Heart Association.

KCCQ: Kansas City Cardiomyopathy Questionnaire (range from 0 to 100).

NT-proBNP: N-terminal pro-B-type natriuretic peptide (NT-proBNP level <1600 pg/mL was considered as normal).

A risk ratio >1 and a *p*-value <0.05 were considered significant.

A *p*-value <0.05 was considered significant.

* Significant factor responsible for recurrent and terminal events after a follow-up period of 18 months.
